# Measurement of sulfatides in the amniotic fluid supernatant: A useful tool in the prenatal diagnosis of metachromatic leukodystrophy

**DOI:** 10.1002/jmd2.12270

**Published:** 2022-01-19

**Authors:** Francyne Kubaski, Zackary M. Herbst, Maira Graeff Burin, Kristiane Michelin‐Tirelli, Franciele B. Trapp, Rejane Gus, Alice B. O. Netto, Ana Carolina Brusius‐Facchin, Sandra Leistner‐Segal, Maria Teresa Sanseverino, Carolina Moura Fischinger de Souza, Matheus V. M. B. Wilke, Thiago Oliveira, Jose A. A. Magalhães, Roberto Giugliani

**Affiliations:** ^1^ PPGBM UFRGS Porto Alegre Brazil; ^2^ Medical Genetics Service HCPA Porto Alegre Brazil; ^3^ INAGEMP Porto Alegre Brazil; ^4^ Department of Chemistry University of Washington Seattle Washington USA; ^5^ PPGCM UFRGS Porto Alegre Brazil; ^6^ Escola de Medicina PUCRS Porto Alegre Brazil; ^7^ Fetal Medicine Unit HCPA, UFRGS Porto Alegre Brazil; ^8^ Department of Genetics UFRGS Porto Alegre Brazil

**Keywords:** arylsulfatase A, metachromatic leukodystrophy, prenatal analysis, sulfatides, tandem mass spectrometry

## Abstract

Metachromatic leukodystrophy (MLD) is an autosomal recessive lysosomal disorder caused by deficiency of arylsulfatase A (ARSA), leading to an accumulation of sulfatides. Sulfatides have been quantified in urine, dried blood spots (DBS), and tissues of patients with MLD. Newborn screening (NBS) for MLD has already been proposed based on a two‐tier approach with the quantification of sulfatides in DBS followed by the quantification of ARSA by liquid chromatography–tandem mass spectrometry (LC–MS/MS). Prenatal screening for MLD is also crucial, and sulfatide quantification in amniotic fluid (AF) can aid diagnosis. The prenatal study was initiated due to a family history of MLD at 19 weeks of gestation. ARSA was quantified in cultured amniocytes. C16:0 sulfatide was quantified by LC‐MS/MS in the supernatant of AF. Molecular analysis of the *ARSA* gene was performed in cultured amniocytes. ARSA was deficient in fetal cells, and C16:0 sulfatides were significantly elevated in comparison to age‐matched controls (3‐fold higher). Genetic studies identified the c.465+1G>A variant in homozygosis in the *ARSA* gene. Our study shows that sulfatides can be quantified in the supernatant of AF of MLD fetuses, and it could potentially aid in a faster and more accurate diagnosis of MLD patients.

**Synopsis**

Sulfatide measurement in the supernatant of AF offers a fast insight into the outcome of the prenatal diagnosis of MLD and can be a useful tool in the overall diagnostic process.

## 1 INTRODUCTION

Metachromatic leukodystrophy (MLD) (OMIM#250100) is a lysosomal disorder caused by deficiency of arylsulfatase A (ARSA), and less frequently by deficiency of saposin B (SapB).^1^, ^2^, ^3^, ^4^ Any of these deficiencies cause impaired catabolism of sulfatides, which accumulate in the central (CNS) and peripheral nervous systems (PNS), leading to demyelination.^4^, ^5^

The clinical signs are related to progressive demyelination and involve deterioration in motor and cognitive functions and behavioral abnormalities. The disease is classified according to the age of onset as late‐infantile (up to 29 months of age), juvenile (30 months to 16 years of age), and adult (after 16 years of age).^3^, ^6^

Ex vivo gene therapy with Libmeldy (Orchard Therapeutics) has been approved for the treatment of MLD in asymptomatic or early‐symptomatic patients in the European Union, while hematopoietic stem cell transplantation (HSCT) is expected to benefit patients with the juvenile form at early disease stages, with conflicting results for other cases.^7^, ^8^, ^9^ A trial with intrathecal enzyme replacement therapy (NCT 03771898) in patients with late‐infantile MLD is currently ongoing.

Early diagnosis of MLD that can be achieved by newborn screening (NBS) via liquid chromatography–tandem mass spectrometry (LC–MS/MS) by a two‐tier approach, which includes quantitation of the C16:0 sulfatide, and assay of ARSA activity in dried blood spots (DBS).^3^, ^10^, ^11^, ^12^

Prenatal identification of MLD can be performed by ARSA quantification and sulfatide measurement in amniocytes or chorionic villus,^6^, ^13^, ^14^, ^15^, ^16^, ^17^ a process that usually takes several weeks. In this study, we report what is, to the best of our knowledge, pioneer use of C16:0 sulfatide quantification in the supernatant of amniotic fluid (AF) and discuss its value for the prenatal diagnosis of MLD.

## 2 MATERIAL AND METHODS

### 2.1 Samples

AF was collected by amniocentesis at 19 weeks and 6 days of gestation. After the baby was born, dried blood spots (DBS), whole blood, and urine were collected for the postnatal confirmation of the prenatal diagnosis.

### 2.2 Culture of amniocytes

Cell culture was performed according to the protocol described by Kessler et al.^18^ In summary, approximately 20 mL of AF was collected and delivered to the laboratory at room temperature. Upon arrival at the laboratory, the AF was centrifuged at 1500 RPM (250 g) for 8 min. The supernatant was collected for C16:0 sulfatide analysis and 4 mL of Gibco AmnioMAX C‐100 complete medium was added to the cell pellet (Thermo Scientific, USA) that was transferred to a 25 cm^2^ culture flask. The flask was incubated at 37°C in a CO_2_ incubator. After 6 days, the first medium change was performed with supernatant removal and the addition of 4 mL of Gibco AmnioMAX C‐100 complete medium. Cell growth and confluence were observed at the inverted microscope and the cells were collected with medium removal and addition of trypsin–EDTA after reaching confluence. The trypsinized cells were centrifuged for 1500 RPM for 8 min. The cells were collected cells and used for ARSA activity quantification and DNA extraction.

### 2.3 Enzyme assays

ARSA activity was quantified in amniocytes by the colorimetric method described in 1987 by Lee‐Vaupel and Conzelman^19^ using a protein concentration of 25 μg/100 μL. The activity of iduronate‐2 sulfatase (IDS) was also quantified to evaluate the quality of the sample and to rule out multiple sulfatase deficiency (MSD).^20^, ^21^ ARSA was quantified in DBS according to the method described by Hong et al.^10^

### 2.4 Sulfatide quantification

C16:0 sulfatide was measured in the supernatant of AF, urine, and DBS by ultraperformance LC–MS/MS (UPLC‐MS/MS) and compared with age‐matched controls. The protocol used for supernatant of AF and urine was slightly modified from Spacil et al.,^11^ and the protocol used for DBS was from Hong et al.^3^ In summary, supernatant of AF or urine (1 nmoles of creatinine equivalents) were lyophilized and one 3.3 mm punch of DBS was incubated with 100 μL of methanol containing 30 nM d5‐C16:0 sulfatide (Gelbchem, Seattle, USA). The samples were incubated at 37°C for 3 h followed by centrifugation at 3000 g for 5 min. Next, 75 μL of the supernatant was transferred to a new 96‐well plate and dried under N2. The samples were reconstituted in 100 μL of 50:50 methanol/water and injected into the UPLC‐MS/MS (Xevo TQ‐S micro, Waters Technologies, USA) by multiple reaction monitoring (MRM) in electrospray ionization in negative mode.

An Acquity UPLC CSH C18 column (1.7 μm, 2.1 mm × 50 mm [part number 186005296], Waters Technologies, USA) was used. Mobile phase A was 70:30 water with acetonitrile with 0.1% formic acid and mobile phase B was 65:35 isopropanol/acetonitrile with 0.1% formic acid. The flow rate was 0.8 mL/min and the gradient was 0.5% B at 0 min, 25% B at 0.75 min, 60% B at 1 min, 75% B at 1.50 min, 100% B at 1.80 min, 100% B at 2.15 min, and 0.5% B at 2.20 min. The MRM was 778.51 > 96.8597 for C16:0 and 783.54 > 96.85 for D5 C16:0. The settings of mass spectrometer source were capillary voltage 3.5 V, source temperature 150°C, desolvation temperature 550°C, and cone gas flow 50 L/h. The results were expressed as μg/mg of creatinine for AF and urine samples and as ng/mL for DBS samples.

### 2.5 Molecular analysis

DNA was isolated from cultured amniocytes using the DNeasy Blood & Tissue Kit (Qiagen, Germany) according to the manufacturer protocol for cultured cells.^22^

Molecular analyses were conducted by next‐generation sequencing (NGS) using Ion GeneStudio S5 (Thermo Scientific) with a customized panel (Ion AmpliSeq Thermo Scientific) including the *ARSA* gene. Data was analyzed on Ion Torrent suite and Ion reporter (Thermo Scientific) version 5.0. The reference sequence used was NM_001085425.2.

## 3 RESULTS

We report the case of a fetus which was the second pregnancy of a nonconsanguineous Brazilian couple (23‐year‐old mother and 25‐year‐old father). The couple's first child was diagnosed with MLD at the age of 3 years, after investigation for a developmental delay (started at 12 months of age) that progressed to neurological regression, epilepsy, and the finding of leukodystrophy in the brain magnetic resonance imaging. Biochemical analyses in the first child showed undetectable ARSA activity in leucocytes (reference range: 5–20 nmol/h/mg of protein); and next‐generation sequencing detected the common pathogenic variant c.465+1G>A (IVS2+1G>A) in the *ARSA* gene in homozygosis.

The second pregnancy, planned after reproductive counseling, occurred naturally and was followed up at a reference center for fetal medicine and metabolic diseases. Amniocentesis for the investigation of MLD was carried out at the gestational age of 19 weeks and 6 days. ARSA activity in cultured amniocytes was 0.35 mmL/h/mg of protein (reference range: 20–50 nmol/h/mg of protein). IDS activity in the same material was normal (68 nmol/4 h/mg of protein [reference range = 50–100 nmol/4 h/mg of protein]), thus excluding MSD.

**FIGURE 1:**
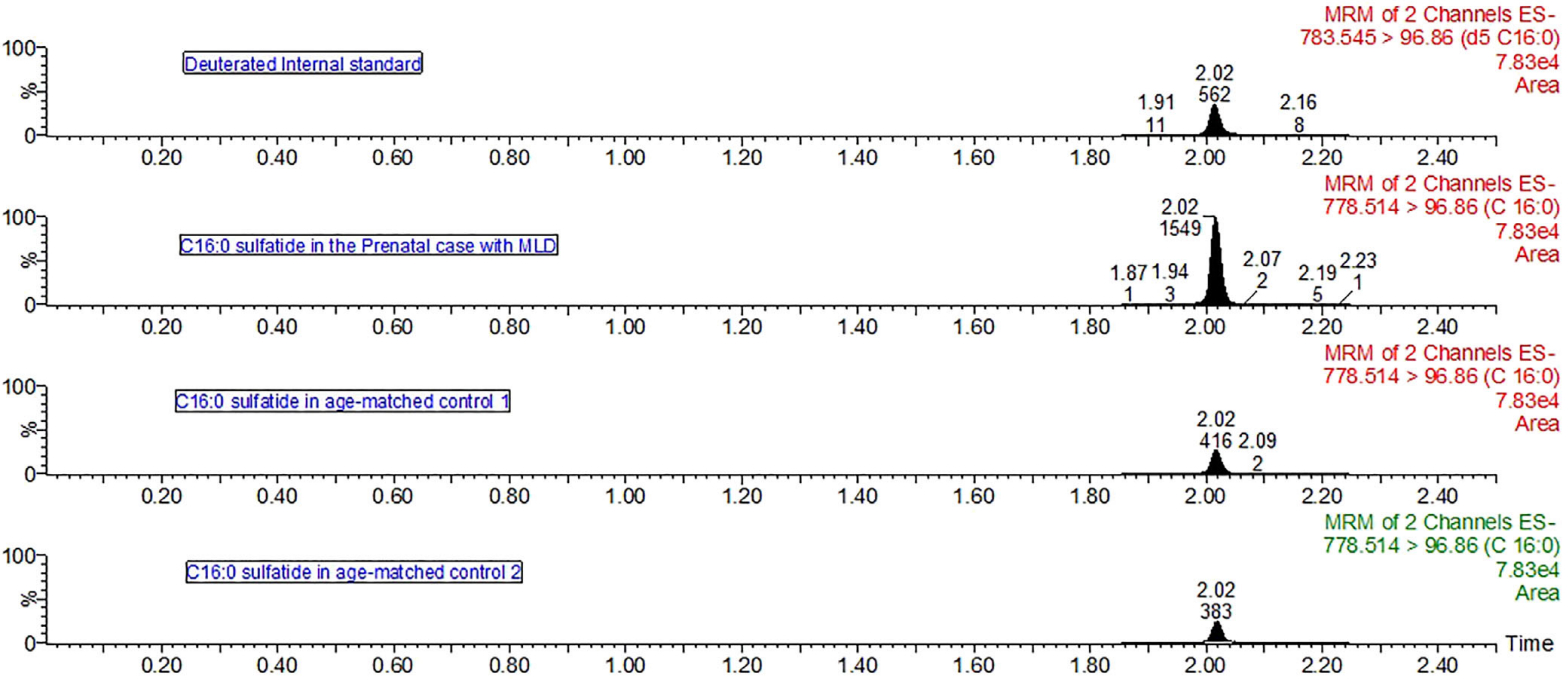
Chromatograms of C16:0 sulfatide in the prenatal case and two age‐matched controls. The top chromatogram in the deuterated internal standard of C16:0; the internal standard confirms the correct retention time of 2.02 min. C16:0 sulfatide quantification in the prenatal case is 4.9 μg/mg of creatinine (gestational age of 19 weeks and 6 days), age‐matched control 1 is 1.4 μg/mg of creatinine (gestational age of 19 weeks and 3 days), and age‐matched control 2 is 1.5 μg/mg (gestational age of 18 weeks)

The quantification of C16:0 sulfatide in the AF supernatant by UPLC‐MS/MS was 4.9 μg/mg of creatinine (range in healthy controls: 1.4–1.5 μg/mg of creatinine) (Figure 1).

Molecular analyses were conducted by NGS with a customized panel including the *ARSA* gene and 34.755 reads were obtained with coverage of 314.2 reads per amplicon. The pathogenic variant c.465+1G>A (IVS2+1G>A) of the *ARSA* gene, for which both parents are heterozygotes, was found in homozygosis in the fetus.

**FIGURE 2:**
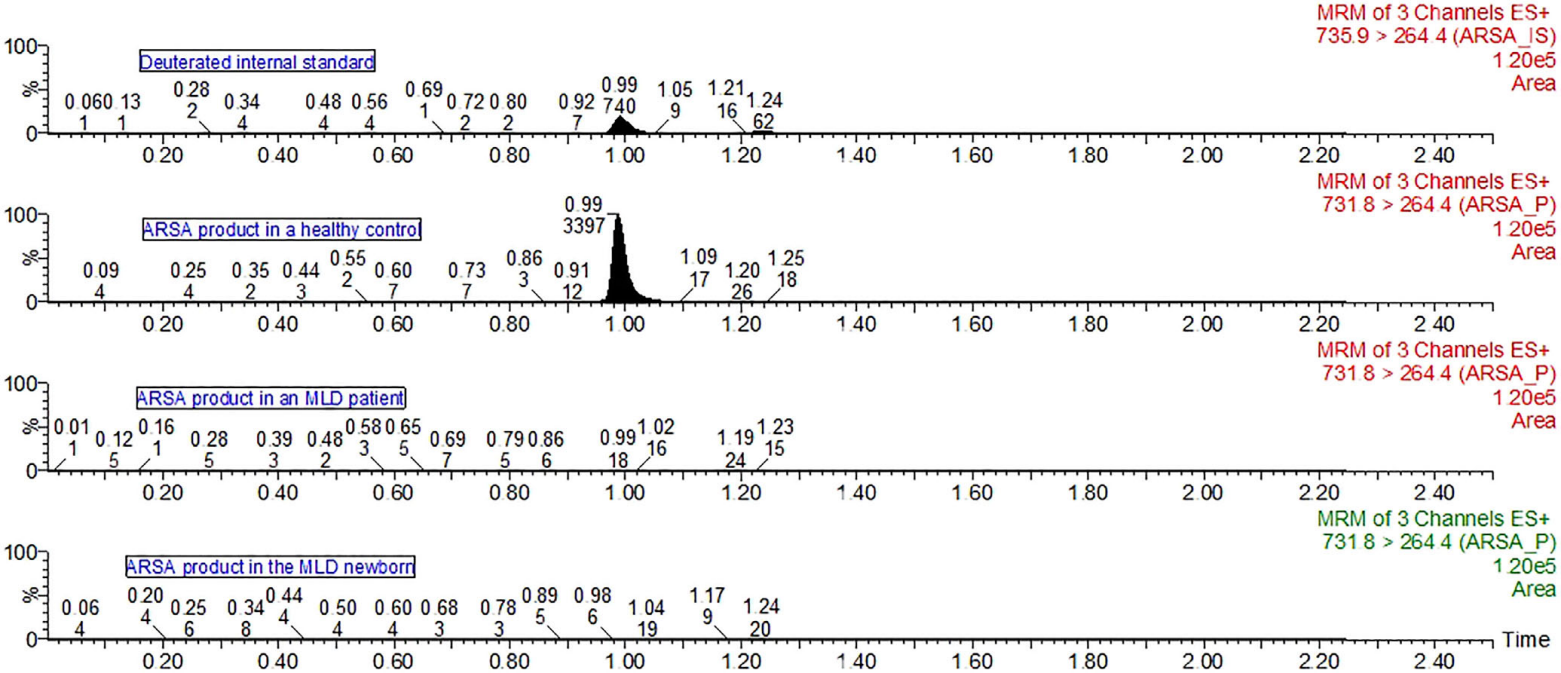
Chromatograms of ARSA internal standard (IS) and product in DBS of the newborn MLD patient, healthy control, and an older MLD patient. The top chromatogram in the deuterated internal standard of ARSA; the internal standard confirms the correct retention time of 0.99 min. ARSA activity in the healthy control was 0.54 μmol/h/L (product area of 3397); ARSA activity in a 4.6‐year‐old MLD patient was 0.01 μmol/h/L (product area of 18); and ARSA activity in the newborn MLD patient was 0 μmol/h/L (product area of 6). The ARSA activity reference range in healthy controls was 0.33–0.76 μmol/h/L. All these values were obtained in DBS

The baby was born by C‐section, with a gestational age of 39 weeks and 4 days. Anthropometry: weight z‐score −0.28, length z‐score −2.05, head circumference z‐score 0.42, and Apgar score 8/9. DBS was collected during the first 24 h of life. ARSA activity was quantified in DBS and was deficient (0 μmol/h/L [reference range in healthy controls = 0.33–0.76 μmol/h/L) (Figure 2).

ARSA activity was also undetectable in the newborn leukocytes (reference range = 5–20 nmol/h/mg of protein). Sulfatide levels were quantified in DBS as 0.14 μg/mL (reference range < 0.13 μg/mL), and sulfatide levels in urine were 0.32 μg/mg of creatinine (reference range < 0.029 μg/mg of creatinine), confirming the increased levels observed in the prenatal analysis. At the age of 5 months, the patients remained asymptomatic, with normal neuropsychomotor development (NPMD). The therapeutic possibilities presently available (gene therapy and HSCT) are being considered.

## 4 DISCUSSION AND CONCLUSIONS

We have demonstrated that a fetus with MLD already has a marked elevation of sulfatides in the AF supernatant at 19 weeks of gestational age. Sulfatide, 3‐O‐sulfogalactosylceramide, is synthesized by ceramide galactosyltransferase (UGT8) and cerebroside sulfotransferase (GAL3ST1) and it is degraded by ARSA. When any of these enzymes is impaired, the sulfatide metabolism is abnormal and it can lead to disease^23^ as sulfatides are essential molecules for several biological functions and tissues such as the nervous system, the immune system, insulin secretion, hemostasis/thrombosis, and infections.^23^ In the CNS, sulfatides are largely found in the myelin sheath and correspond to 4% of total myelin lipids.^24^ They have several major functions in the CNS and PNS such as: acting as a negative regulator of oligodendrocyte differentiation,^25^ oligodendrocyte survival,^26^ initiation factor of myelin synthesis of Schwann cells,^27^ inhibition of myelin‐associated axon outgrowth,^28^ glial‐axon signaling,^29^ and myelin maintenance.^23^, ^29^ Therefore, impaired sulfatide metabolism can lead to severe and irreversible manifestations.

MLD is a lysosomal storage disease caused by genetic variants that lead to deficiency of ARSA or SapB affecting sulfatide metabolism. Thus, it is characterized by progressive damage of the myelin sheath of the CNS and PNS. The disease severity depends on the residual enzyme levels that are related to the type of variants.^6^

As treatment measures are becoming available, the early diagnosis of MLD is becoming critically important, as early treatment will be required to prevent progressive demyelination. NBS initiatives will help to increase treatment efficacy and to positively modify the natural history of the disease.^3^, ^6^, ^30^ It is very important to discriminate true MLD from carrier status and pseudodeficiency. In addition, variants of unknown significance (VUS) and pseudodeficiency variants that have not been described a challenge the diagnostic elucidation, and it can be a pitfall in the diagnosis of MLD.^6^

Thus, sulfatide quantification is a highly useful biomarker in the diagnostic process of MLD.^3^, ^10^, ^11^ It has been used as an important biomarker in prenatal diagnosis, NBS, and diagnosis of older patients with MLD.^3^, ^6^, ^10^, ^11^ In our experience, sulfatide quantification has also helped to differentiate MLD from pseudodeficiency and to better understand the significance of genetic variants classified as VUS.

Prenatal diagnosis of MLD is usually performed by enzyme quantification and/or molecular analysis. To our knowledge, this is the first report of quantification of C16:0 sulfatide by UPLC‐MS/MS in the supernatant of AF from an MLD fetus, confirming that sulfatide accumulation starts already very early in life. This is expected in a patient who is homozygous for the variant c.465+1G>A, which has been associated with the onset of clinical manifestations before 30 months of age in most affected patients.^31^

In the present case, the measurement of C16:0 sulfatide in the AF supernatant provided an early insight on the outcome of prenatal diagnosis in a pregnancy at risk for MLD and helped the team to provide the best possible approach to the parents along the process, proving to be a helpful tool in the overall prenatal diagnosis process.

## CONFLICT OF INTERESTS

Francyne Kubaski, Maira Graeff Burin, Kristiane Michelin‐Tirelli, Franciele B. Trapp, Rejane Gus, Alice B. O. Netto, Ana Carolina Brusius‐Facchin, Sandra Leistner‐Segal, Maria Teresa Sanseverino, Carolina Moura Fischinger de Souza, Matheus V. M. B. Wilke, Thiago Oliveira, Jose A. A. Magalhães, Roberto Giugliani declare that they have no conflict of interest. Zackary M. Herbst is a consultant for GelbChem, LLC.

## INFORMED CONSENT

The study was conducted according to the guidelines of the Declaration of Helsinki, and approved by the Institutional Review Board (or Ethics Committee) of HCPA (protocol code 2006‐0351 on October 2006). Informed consent was waived by the IRB because this testing is part of the diagnostic workflow of the IEM Brazil Network.

## Data Availability

Data archiving is not mandated but data will be made available on reasonable request.
